# Correction: Azithromycin consumption during the COVID-19 pandemic in Croatia, 2020

**DOI:** 10.1371/journal.pone.0272324

**Published:** 2022-07-26

**Authors:** Nikolina Bogdanić, Loris Močibob, Toni Vidović, Ana Soldo, Josip Begovac

The fifth author’s name is spelled incorrectly. The correct name is: Josip Begovac. The correct citation is: Bogdanić N, Močibob L, Vidović T, Soldo A, Begovać J (2022) Azithromycin consumption during the COVID-19 pandemic in Croatia, 2020. PLoS ONE 17(2): e0263437. https://doi.org/10.1371/journal.pone.0263437
