# Supplementary material for: Azithromycin consumption during the COVID-19 pandemic in Croatia, 2020
Source: PLoS One. 2022 Feb 2;17(2):e0263437. doi: 10.1371/journal.pone.0263437 (PMC8809604; doi:10.1371/journal.pone.0263437)
Supplement: S1 File — (DOCX) [file pone.0263437.s001.docx]

**
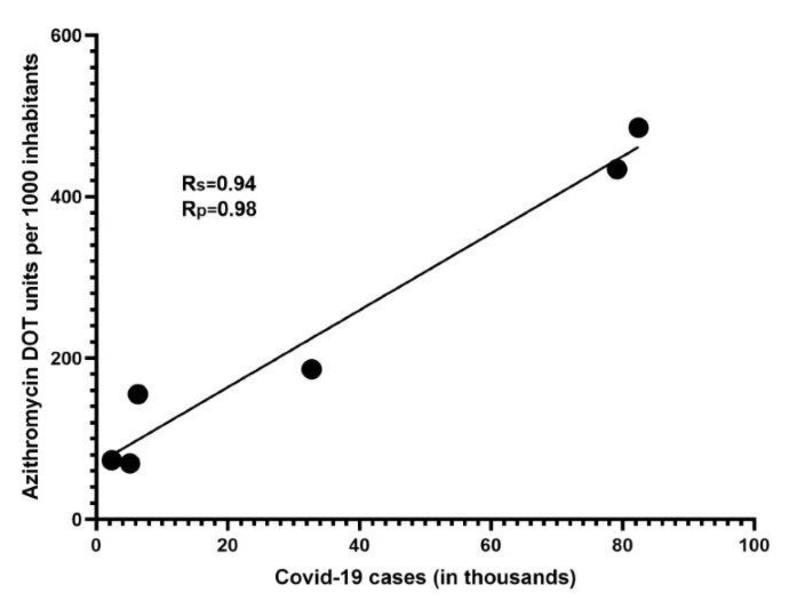
**

**Figure A.** The relationship of COVID-19 cases and total azithromycin distribution (hospital and non-hospital pharmacies) in Croatia from July (first dot) to December 2020 (last dot). DOT, days of therapy

**Table A.** Total azithromycin distribution to hospital and non-hospital pharmacies by quarter in Croatia, January 2017 to December 2020.^a^

|  | Quarters | | | |
| --- | --- | --- | --- | --- |
|  | January - March | April - June | July - September | October - December |
| Monthly mean in 2020 | 23.6 | 2.2 | 4.9 | 18.2 |
| Monthly mean  for 2017-2019 | 16.7 | 8.5 | 8.2 | 11.8 |
| Difference (95% CI) | 6.9 (-6.2 to 20.1) | -6.3 (-7.6 to -5.0) | -3.3 (-8.2 to 1.5) | 6.4 (-12.8 to 25.5)^b^ |
| Difference, % change 2017-2019 vs 2020 | 41.6 | -73.8 | -40.5 | 54.0 |
| P-value | 0.217 | <0.001 | 0.128 | 0.297 |

^a^ Data are presented as number of 1500 mg doses distributed per 1000 inhabitants (population numbers were extracted from Eurostat). ^b^ The Satterthwaite method was used because the F-test for equality of variances was rejected. CI, confidence interval.
