# Supplementary material for: Azithromycin consumption during the COVID-19 pandemic in Croatia, 2020
Source: PLoS One. 2022 Feb 2;17(2):e0263437. doi: 10.1371/journal.pone.0263437 (PMC8809604; doi:10.1371/journal.pone.0263437)
Supplement: Attachment — Submitted filename: rebuttal letter 12.11..docx [file pone.0263437.s002.docx]

We wish to appeal the decision on the manuscript „Azithromycin consumption during the COVID-19 pandemic in Croatia”, 2020 PONE-D-21-11999R3.

A point-by-point response to the Editors’ comments, which are repeated in italics, is given below.

***Overall scoring (1-5)*

*1. Novelty: 3*

*2. Writing: 4*

*3. Methodology: 2*

*4. Data presentation: 3*

*5. Data interpretation: 3*

We strongly disagree with the judgement that our manuscript is not methodologically sound. The statistical analysis in our manuscript is simple and follows the usual presentation of such data published in other studies. Namely the main focus of the analysis was the comparison of drug consumption (in this case azithromycin) in a per year or monthly interval in one year (2020 in this case) to the consumption of the same drug in the same period to previous years (2017, 2018, 2019). Our main analysis (see the first paragraph of the results section) was focused on the increase of azithromycin consumption expressed in percentages (“This was a 5.2% increase from 2019 to 2020 and an 8.1% increase from the average of 2017–2019 to 2020. This 8.1% increase corresponds to a total of 37224 5-day courses of azithromycin prescriptions.”). We also focused on how many times more azithromycin was prescribed (see abstract: “Non-hospital pharmacies received 2.18 times and hospital pharmacies 4.39 times more DOT units/1000 inhabitants of azithromycin in March 2020 compared to the average distribution rate in March 2017–2019.") During the peak of the COVID-19 epidemic (November and December 2020) azithromycin distribution increased considerably in hospitals (3.62 and 3.19 times, respectively) and non-hospital pharmacies (1.93 and 1.84 times, respectively) compared to the average consumption in the same months in 2017–2019.)” We also presented our data in a figure and the monthly distribution of the consumption of azithromycin in 2020 and was very different than in years 2017-2019 (see figure). So, our main finding does not depend on the Spearman correlation analysis.

*** Statistical analysis: A investigator needs to examine three assumptions to run*

*Spearman's Rank-Order Correlation test.*

*1. Type of variables: ordinal, interval, or ratio scale*

*2. Paired observation*

*3. Monotonic relationship*

*In this study, the 2nd assumption is not considered. And, the 3rd one is not addressed.*

In the context of the monthly distribution from July to December 2020 we compared the absolute number of COVID-19 cases from July to December 2020 with the total azithromycin consumption (the definition of consumption in clearly stated in the method section “Azithromycin consumption was measured as days of therapy (DOT) which represents a ratio of the total dose of azithromycin in one package divided by the defined daily dose unit of 0.3 gr.”). By examining the figure it can be concluded that all assumptions for the Spearman correlation coefficient were met (this seems to be clear to the first reviewer). The variables are continuous. The data is paired by months (the number of COVID-19 cases in July are paired with the consumption of azithromycin in July, and the same is paired for August, September, October, November and December), so this is a paired sample. The figures also show that the increase in COVID-19 cases is correlated with the increase in azithromycin consumption in the period from July to December 2020. Hence the assumption of monotonic relationship is met (“In a monotonic relationship, the variables tend to move in the same relative direction, but not necessarily at a constant rate”… “Linear relationships are also monotonic” see for example [https://support.minitab.com/en-HYPERLINK "https://support.minitab.com/en-us/minitab/19/help-and-how-to/statistics/basic-statistics/supporting-topics/basics/linear-nonlinear-and-monotonic-relationships/"us/minitab/19/help-and-how-to/statistics/basic-statistics/supporting-topics/basics/linear-nonlinear-and-monotonic-relationships/](https://support.minitab.com/en-us/minitab/19/help-and-how-to/statistics/basic-statistics/supporting-topics/basics/linear-nonlinear-and-monotonic-relationships/)). Enclosed to our manuscript as S1 Supporting Information are graph (Figure A) and table with original values (Table A) further supporting the monotonic relationship of COVID-19 cases and azithromycin consumption. So, all requirements to perform Spearman’s correlation are met.

***- This study has tried to rule out the effect of other factors influencing the rate of macrolide consumption by referring to studies of other countries (for example, coinfection to a study from North California, or Influenza to a general report from Europe). To make the results more reliable, the investigators need to evaluate and rule out the other reasons for azithromycin overuse during the pandemic IN CROATIA.*

Our analysis was not around “ruling out other factors influencing the rate of macrolide consumption”. We simply stated in our discussion that we did not have an influenza epidemic during the winter months of 2020 and that coinfection with other pathogens in COVID-19 is rare. It is well known that during the influence epidemic there is an increase of antibiotic consumption. We provided data of azithromycin distribution in Croatia for the previous three years (2017-2019) and it showed that the monthly pattern of azithromycin distribution was similar in 2017, 2018 and 2019 and completely different in the COVID-19 year (2020). The suggestion of the editorial review that there might have been other causes (other than COVID-19) of increase azithromycin consumption in Croatia in 2020 compared to previous 3-years is very unlikely and this has been now discussed in our paper. Furthermore, the three authors (NB, LM, JB) of the manuscript work at the only special 220-bed (exclusively) infectious diseases hospital in the capital of Croatia, Zagreb. At the beginning of the COVID-19 epidemic in Croatia (March 2020), there was a decision of the management of the hospital to purchase large quantities of azithromycin for treatment of COVID-19 because of speculations that azithromycin might help in the treatment of COVID-19. At that time and afterwards in 2020 there was no other infectious disease epidemic going on in Zagreb nor in Croatia that would need such large quantities of azithromycin for treatment. This (azithromycin overconsumption) seems to have happened in other countries and settings, as the first reviewer alluded to (Dr Iwein Gyselinck stated “I compliment the authors with the well written article, providing insight in azithromycin use, *something many suspect but few have published upon*”).

Moreover, healthcare professionals in Croatia are obligated to report 92 different infectious disease entities including all forms of pneumonia (including those caused by *M. pneumoniae*, *Chlamydia*, etc; document in Croatian: <https://narodne-novine.nn.hr/clanci/sluzbeni/1994_03_23_410.html>) to the Croatian Institute of Public Health. There was no report from the Croatian National Public Health Institute of a large number of non-Covid pneumonias occurring in the whole 2020. It would be highly unlikely that during the outbreak of COVID-19 we simultaneously had a large outbreak of another infectious disease that is treated by azithromycin. We added two sentences to our original manuscript in the discussion section: “One may also speculate about other non-COVID-19 causes of azithromycin overconsumption in 2020. However, this is very unlikely as there was no report from the Croatian National Public Health Institute of any other ongoing epidemic requiring azithromycin treatment in 2020.”

We also received two suggestions from the editor: *1. to add the information of azithromycin adverse effects during the pandemic and 2. to compare the covid-19 mortality based on a rate of azithromycin administration.* However, we did not analyse individual data so it is impossible to address those two issues in a meaningful way. Also, the aim of our study was not to assess factors related to COVID-19 mortality, so this analysis on the data we have would not give a valid result.

Finally, as we consider this topic needs prompt attention of healthcare workers who have a role in treatment of COVID-19 patients we posted our manuscript on preprint server medRxiv on 1st of November 2021: <https://medrxiv.org/cgi/content/short/2021.10.31.21265714v1>.

Thank you for giving us the opportunity to respond. We look forward to hearing from you in a reasonable time. We would be glad to respond to any further questions and comments that you may have.

On behalf of all authors,

Nikolina Bogdanić and Josip Begovac
