# Supplementary material for: Azithromycin consumption during the COVID-19 pandemic in Croatia, 2020
Source: PLoS One. 2022 Feb 2;17(2):e0263437. doi: 10.1371/journal.pone.0263437 (PMC8809604; doi:10.1371/journal.pone.0263437)
Supplement: Attachment — Submitted filename: review_PLOS ONE.docx [file pone.0263437.s003.docx]

**Comments**

- To study the correlation authors can modify the plot by taking independent variable (No of Covid -19 cases) on X- axis and dependent variable (Azithromycin usage) on Y- axis as per below (reference plot). Exclude the monthly distribution data for this analysis. Graph representing monthly distribution can still be retained and explained separately.

r_p_ =Pearson’s product moment correlation coefficient_,_ r_s_ = Spearman’s rank correlation coefficient

Spearman correlation is employed when two variables are monotonically related. Which means all the data should be entirely in an increasing trend or in a decreasing trend and not a combination of both. The present data doesn’t strictly follow a monotonous relation neither a linear relation (Azithromycin usage in the month of march is an outlier)

Authors can explore qualities of both correlation test (Pearson’s product moment and spearman’s rank correlation) and write the discussion accordingly

For instance, spearman’s correlation explains the strength of monotonous relation, so the explanation for this could be how strict the data was in terms of monotonous relation (every increase in Covid-19 case resulting in increase of azithromycin usage)

Pearson’s correlation explains strength of linear correlation, so the explanation for this could be if the rate of increase in the Azithromycin usage was constant for every increase in Covid-19 case

- Comparison between monthly usage of Azithromycin for the year 2020 and the average use of azithromycin for the period 2017-2019 could be tested for significance. Authors can use ANOVA pair wise comparison and rate the difference as per p-value which can be denoted in graph using asterisks
