# Supplementary material for: Azithromycin consumption during the COVID-19 pandemic in Croatia, 2020
Source: PLoS One. 2022 Feb 2;17(2):e0263437. doi: 10.1371/journal.pone.0263437 (PMC8809604; doi:10.1371/journal.pone.0263437)
Supplement: Attachment — Submitted filename: rebutal letter 6.1..docx [file pone.0263437.s004.docx]

**Azithromycin consumption during the COVID-19 pandemic in Croatia, 2020**

**Response to Reviewers**

We are grateful to the reviewers for the comments on our paper. Enclosed herein is the revised version of our manuscript with supplemental material (S1 Supporting Information). A point-by-point response to the Reviewers’ comments, which are repeated in italics, is given below.

Firstly, we would like to emphasize that the main focus of the analysis in our study was to describe the distribution of azithromycin to the hospital and non-hospital pharmacies in the years 2017 to 2020. Azithromycin distribution per months to non-hospital and hospital pharmacies was planned to be presented graphically. Our main analysis was focused on the difference in azithromycin consumption expressed in percentages. So, our main findings do not depend on the correlation analysis.

*Reviewer #2: Comments
• To study the correlation authors can modify the plot by taking independent variable (No of Covid -19 cases) on X- axis and dependent variable (Azithromycin usage) on Y- axis as per below (reference plot). Exclude the monthly distribution data for this analysis. Graph representing monthly distribution can still be retained and explained separately. Spearman correlation is employed when two variables are monotonically related. Which means all the data should be entirely in an increasing trend or in a decreasing trend and not a combination of both. The present data doesn’t strictly follow a monotonous relation neither a linear relation (Azithromycin usage in the month of march is an outlier)
Authors can explore qualities of both correlation test (Pearson’s product moment and spearman’s rank correlation) and write the discussion accordingly
For instance, spearman’s correlation explains the strength of monotonous relation, so the explanation for this could be how strict the data was in terms of monotonous relation (every increase in Covid-19 case resulting in increase of azithromycin usage)
Pearson’s correlation explains strength of linear correlation, so the explanation for this could be if the rate of increase in the Azithromycin usage was constant for every increase in Covid-19 case.*

Spearman’s rank-order correlation test was used to measure the association between quantities of azithromycin distribution and the number of COVID-19 cases **from July to December 2020**. There was a positive correlation between the number of COVID-19 cases and the total azithromycin consumption from July to December 2020 (ρ=0.94, p=0.005). July 2020 was the first month we included in this analysis due to the obvious increase in number of COVID-19 cases (531 cases in June, 2361 case in July).

We are a bit puzzled by the statement of the reviewer “Spearman correlation is employed when two variables are monotonically related. Which means all the data should be entirely in an increasing trend or in a decreasing trend and not a combination of both.” If this means that a monotonic relationship is a prerequisite for performing the Spearman rho we disagree. If this is not the case, we apologize for the explanation on why a monotonic relationship is not a prerequisite for performing the Spearman test.

We have not been able to confirm that a monotonic relationship is a prerequisite for performing the Spearman test by looking at statistical textbooks (e.g. Armitage, Berry, Matthews: Statistical Methods in Medical Research ed 4 2002. A. Petrie and Sabin C. Medical statistics at a Glance 3rd edition 2009) nor does an online textbook <https://statistics.laerd.com/statistical-guides/spearmans-rank-order-correlation-statistical-guide.php> mention this. On the contrary, on the Laerd statistics web site we can find the following sentence “A monotonic relationship is not strictly an assumption of Spearman's correlation. That is, you can run a Spearman's correlation on a non-monotonic relationship to determine if there is a monotonic component to the association.” The question on the “monotonic assumption” of Spearman’s test appeared at least 3 times in the statsexchange forum (see <https://stats.stackexchange.com/questions/109793/spearmans-monotonic> ; <https://stats.stackexchange.com/questions/161376/definition-of-monotonic-and-implications-for-using-spearmans-correlation> ; <https://stats.stackexchange.com/questions/149350/how-to-identify-that-relationship-between-two-variables-are-monotonic-or-not>). All responses mentioned that a monotonic relationship is not a prerequisite for performing Spearman’s test because the test is testing whether a monotonic relationship is present or not or how strong it is. One of the commentators mentioned that it would be “strange and not true” to “test only for a monotone relationship if you already know there's a monotone relationship." This question also came up at Research Gate: (<https://www.researchgate.net/post/Can_I_run_spearmans_correlation_on_non_monotonic_data_Is_it_advisable>) and again the answer of several commentators was in the line: “Yes, there is nothing wrong with using Spearman correlation coefficient” (on non-monotonic data). Specifically, the quoted comment was provided by Abolfazl Ghoodjani, who is presently professor of statistics at the Mc Gill University, Montreal Canada.

As suggested by Reviewer and Editor, we included additional analyses as supplemental material (S1 Supporting Information) and included comments about it in the Methods and Discussion. We provided a graph on the Spearman-rho which was calculated in our manuscript. We also provided additional statistical analysis but would like to stress again that this study was not planned as a hypothesis-driven study. We simply wanted to present descriptive data on azithromycin distribution between 2017 to 2020. We present our main data in absolute numbers of DOTs based on 1000 inhabitants-days of azithromycin distribution. The comparisons between years are reported as percent or rate difference. The monthly DOTs are reported per 1000 inhabitants and presented graphically. Since this seems not to be clear we rewrote the Methods section and included what our main analysis was about. We believe that the Spearman rho and the additional statistics should not be the main focus of our study and they contribute little to our main conclusion. This is why we put them into the Supplemental material.

*• Comparison between monthly usage of Azithromycin for the year 2020 and the average use of azithromycin for the period 2017-2019 could be tested for significance. Authors can use ANOVA pair wise comparison and rate the difference as per p-value which can be denoted in graph using asterisks*

Thank you for this comment. The additional statistics includes a t-test because we compare only two groups (one way ANOVA and t-test are equivalent with 2 groups). We would like to point out that we do not have individual (daily or weekly data). We have only total DOTs for each month for the period 2017 to 2020. So, for example, we cannot compare by ANOVA January 2020 to anything else because we have only one number for January 2020. We included into the revised manuscript a comparison of the mean monthly dispension of azithromycin by quarters. In this analysis the number of observations in the mean monthly quarterly comparison is small (e.g. 3 in the group 2020 *versus* 3 in the group 2017-2019), so the test has little power to detect a difference. Therefore, it is not surprising that we do not have a significant P in the 1^st^ and 4^th^ quarter. Also, there is no significant difference in the monthly distribution for all 12 months when 2020 is compared to the average of 2017 to 2019 (and yes the variances are not equal). But does this formal statistical analysis of the mean monthly comparison for the whole year make sense if you look at the pattern of monthly distribution (see graphs)? Also, the nonsignificant P-values derived from the above-mentioned analyses should not be interpreted as absence of overconsumption of azithromycin in 2020. Almost 38 000 extra 5-day courses of azithromycin were distributed in 2020 compared with previous years, with no obvious other reasons but COVID-19 which is quite relevant despite a „nonsignficant“ P. Of note, COVID-19, as discussed in our paper, should not be treated with azithromycin. Let’s not loose common sense when interpreting data.

*Reviewer #3: The article looks interesting, not very hard earned statistical paper but good evidence is present. the study is not a part of dual publication. the concern in the science is good enough*

We thank for this comment. We don't think that every paper need hypothesis-driven inferential statistics if enough evidence is present to draw conclusions from the description of the data. Moreover, similar studies published in high-quality journals did not report any P-values and were based on description (for example: King LM et al. Trends in U.S. outpatient antibiotic prescriptions during the COVID-19 pandemic. Clin Infect Dis. 2020 Dec 29. doi:10.1093/cid/ciaa1896). We agree with the reviewer that “good evidence is present”.

We consider this topic needs prompt attention of healthcare workers who have a role in treatment of COVID-19 patients to consider more careful antimicrobial prescribing. We appreciate the valuable time of all Reviewers and Editors and also all suggestions they had so far but we must say that the period of almost 9 months for evaluation of this manuscript is quite long. Our original submission was rejected based on the wrong judgment of the Editor in charge that the Spearman test was inappropriately applied to our data. Please note there is nothing wrong with the Spearman test in our analysis and, in addition, the main result of our study does not even depend on the result of Spearman’s rho. The other reviewer of our original submission (Dr Iwein Gyselinck), similarly to the one of the current reviewers, also concluded that the methods used for reaching our conclusion are appropriate and sound and had no issues regarding the use of Spearmans test. Dr Iwein Gyselinck, an expert in the field of respiratory diseases, recommended publishing our paper.

We thank you for your reevaluation of our manuscript and consideration for publication.

On behalf of all authors,
Nikolina Bogdanić and Josip Begovac
